# Molecular Diagnostics in the Times of Surveillance for *Candida auris*

**DOI:** 10.3390/jof5030077

**Published:** 2019-08-20

**Authors:** Milena Kordalewska, David S. Perlin

**Affiliations:** Center for Discovery and Innovation, Hackensack Meridian Health, Nutley, NJ 07110, USA

**Keywords:** *Candida auris*, diagnostics, molecular diagnostics, identification, detection, antifungal drug resistance, azoles, echinocandins

## Abstract

Recently, global health professionals have been significantly challenged by the emergence of *Candida auris* and its propensity to colonize human skin, persist in the healthcare environment, and cause healthcare-associated outbreaks. Additionally, *C. auris* isolates are often characterized by elevated minimal inhibitory concentration (MIC) values for antifungal drugs. Thus, rapid detection and accurate identification of *C. auris* together with an assessment of potential antifungal drug resistance has become essential for effective patient management, and infection prevention and control in healthcare facilities. Surprisingly, almost all of the commonly available diagnostic tools rely on recovery (growth) of yeast colonies from collected samples, which delays the diagnostic result by several days or longer. To circumvent these issues, molecular-based DNA amplification assays have been developed to identify *C. auris* DNA directly from patient samples. Moreover, allele discriminating detection probes can be used to rapidly assess validated mechanisms of echinocandin and azole resistance.

## 1. Surveillance for *Candida auris*

*Candida auris* is an emerging, multidrug-resistant yeast that causes invasive infections, and has been associated with outbreaks in healthcare settings. The first isolate of *C. auris*, recovered from the external ear canal discharge, was reported in 2009 [1]. Moreover, a South Korean bloodstream isolate collected in 1996 (at that time categorized as an unidentified yeast) was incidentally identified by molecular techniques in 2011 [2]. Since then, many cases of bloodstream and other types of infections have been increasingly reported from multiple countries located on all inhabited continents [3]. Limited data indicate that the highest risk of developing *C. auris* infection is associated with prolonged hospital and long-term care facility stays (critically ill patients, with serious underlying medical conditions and significant healthcare exposure), placement of central venous catheter and/or tubes (breathing, feeding etc.), use of broad-spectrum antibiotic and antifungals, as well as receiving medical care in facilities where cases of *C. auris* infection or colonization were reported [4].

In contrast to most *Candida* species, where antifungal drug resistance is the exception, *C. auris* isolates are often characterized by elevated minimal inhibitory concentration (MIC) values for azoles, polyenes, and echinocandins [5]. Almost universal, high-level resistance to fluconazole is observed in *C. auris* isolates from India and South Africa [6,7,8,9]. Reports of elevated amphotericin B MIC values are not as common as for fluconazole, and resistance rates vary between studied *C. auris* isolates populations (0–30%) [6,9,10,11,12,13]. First line therapy drugs, echinocandins, seem to be the most effective against *C. auris* isolates; however echinocandin-resistant isolates have been identified [9,14,15,16,17].

A unique feature of *C. auris*, differentiating it from other *Candida* species, is the ability to persist on human skin and in the healthcare environment, which results in spread within and across healthcare networks. Chow et al. performed a country-wide whole genome sequencing-based molecular epidemiological survey, assessed the genetic similarity between the isolates from the same patients, healthcare facilities, and states, and discovered local and ongoing transmission in the United States [18]. In New York City, epidemiologic links indicated a large, interconnected web of affected healthcare facilities throughout the city [19]. Similar patterns have been observed in other countries including Colombia [13], Spain [20], and the United Kingdom [21]. All this is very different from typical *Candida* behavior and resembles the spread of multidrug-resistant bacteria [22,23].

Little is known about community transmission of *C. auris*. Patients may be asymptomatically colonized with *C. auris* on the skin, nares, oropharynx, rectum, and other body sites. Jackson et al. speculated that community transmission is often undetected since colonized individuals are not at risk for invasive infection. However, transmission promotion happens in healthcare environments e.g. the ones which provide long-term high-level care to severely ill or disabled patients, especially if infection prevention and control measures are inadequate [24]. Simply, individuals “just colonized” can disseminate *C. auris*, which can reach the ones at risk for invasive infections. Thus, screening patients for *C. auris* colonization is an important component of surveillance for *C. auris* since it allows for identification of colonized individuals and implementation of infection prevention and control measures.

It is recommended to sample one or more high-yield body sites with a swab to identify individuals colonized with *C. auris*. Results of comprehensive studies, which included swabbing of multiple sites (nares, ears, oropharynx, axilla, groin, and rectum) showed that screening with a composite swab should be performed in the bilateral axillae and groin (approximately 90% of cases positive by swab). Additional body sites, including nares (second most positive body site), may be swabbed if feasible. Swabs processing needs to be coordinated through local or state health departments and CDC since it is not commercially supported yet [25]. 

To date, nearly all reported colonization cases have been among patients in healthcare facilities. Additional studies, which will improve our understanding of the prevalence and duration of colonization in the broader population and community transmission, are needed. In fact, as we learned by dealing with multidrug-resistant bacteria, early and aggressive approach to control the newly emerged organism is more effective and efficient in controlling transmission than later response to a widespread problem [25,26,27]. Seconding this approach, Jackson et al. have called for a global response to contain the spread of *C. auris* infections by developing effective disinfection methods, interventions to reduce patient colonization, rapid tests for detecting patient colonization (to target infection control measures), and broader detection capacity across the globe in clinical laboratories [24]. Rhodes and Fisher also underlined the need for the development of global surveillance tools that would facilitate outbreak response [3]. All these efforts require local and international collaboration between all involved stakeholders, including healthcare employees (physicians, nurses etc.), infection prevention and control specialists, public health officials, researchers, and industry.

Here, we argue that new molecular methods perfectly fill the gaps in *C. auris* surveillance methodology and can substantially enhance detection capacity and facilitate outbreak response.

## 2. Detection and Identification of *Candida auris* in Surveillance Samples

### 2.1. Standard Processing of Surveillance Samples

Salt Sabouraud Dulcitol enrichment broth protocol has been developed to facilitate isolation of *C. auris* from surveillance samples (clinical skin swabs and environmental sponges) and is currently followed by the CDC, regional, and state public health laboratories in the U.S. The enrichment for *C. auris* in the broth is obtained by the use of dulcitol instead of dextrose as the main carbon source, which decreases (but not completely excludes) growth opportunities of other, non-target species like *C. glabrata* and *C. parapsilosis* [28]. The protocol requires incubation of aliquots of swab medium inoculated into enrichment broth at 40 °C (temperature selective for *C. auris*) and monitoring of samples turbidity daily for several days or even weeks [29]. Every sample has to be plated onto CHROMagar Candida and screened for the presence of beige, pink, white, and sectored (dark purple) colonies [30]. Recovered colonies have to be further identified to the species level by one of the reliable identification methods, usually MALDI-TOF MS or rDNA sequencing. Applicability of standard biochemical identification systems, including VITEK 2, BD Phoenix, and API 20C, is limited since *C. auris* has often been misidentified by them [22].

The slow turnaround time of enrichment cultures is a major limiting factor in the diagnosis of *C. auris* colonization. Generated delay prevents rapid infection control actions which are of utmost importance taking into account the transmission properties of this yeast. Moreover, depending on the scale of surveillance sampling, laboratories may have to perform enrichment cultures for tens or hundreds swabs each week (as soon as possible after the delivery of samples, since the viability of *C. auris* cells in swabs decreases with time [31]) what is often on the verge or even beyond their capabilities (taking into account that they have to maintain their regular operations and not to switch to exclusive *C. auris* screening). Increased workloads can also happen at the stage of selecting colonies for identification. Colonies of other species, especially *C. parapsilosis*, can also be recovered on CHROMagar Candida and appear the same as *C. auris* colonies [31].

MALDI-TOF MS is best suited for high-throughput identification of *C. auris* from colonies recovered in the enrichment broth protocol, given a complete panel of *C. auris* reference spectra is present in the database [32,33,34]. MALDI-TOF MS analysis is characterized by short preanalytical procedure (sample preparation) and turnaround (results generation in an automated analytical procedure) times, and low running costs [35]. Accurate identification of *C. auris* and differentiation from other yeast can also be obtained by sequencing of PCR-amplified rDNA genetic loci (internal transcribed spacer and D1/D2 region of large subunit (LSU)) [36]. However, sequencing may not be the first choice for routine determination of fungal species in some laboratories, mostly due to its technical demands, and longer turnaround times [22]. Moreover, a potential limitation for broad application of both MALDI-TOF MS and sequencing is the requirement of substantial upfront investment (instrument purchase).

### 2.2. Application of Molecular Diagnostics for Surveillance Sample Processing

The issues resulting from the requirement of culture for MALDI-TOF or rDNA sequencing, widely discussed in the previous paragraph, have created an increasing demand for new diagnostic methods that would enable *C. auris* detection in surveillance swabs in much shorter time. Molecular-based methods have an incomparable potential of being the most efficient tool for high-throughput screening of surveillance samples. If properly validated, they can deliver the diagnostic result within several hours, since the DNA can be isolated directly from the patient specimen without the need of obtaining a colony. Moreover, they can be introduced in places where MALDI-TOF MS or sequencing equipment is not affordable due to financial constraints. 

Given how “hot” the *C. auris* topic is, it is not surprising to see that many research groups (academic and commercial) have developed a variety of molecular-based assays for the identification of *C. auris*:

- PCR and/or real-time PCR [37,38,39,40,41,42,43,44,45,46];
- T2 magnetic resonance [47];
- Loop-mediated isothermal amplification (LAMP) [48].

Most of the developed assays are characterized with the detection limits in the range of 1–10 *C. auris* CFU/PCR reaction, which assures high sensitivity since *C. auris* burdens on patient skin swabs are much higher, with up to 10^8^ CFU on a single swab [49]. In many cases, specificity of the assays was confirmed by testing large panels of closely and distantly related yeasts and other pathogens, to assure accurate species-level identification. So far, SYBR Green [29] and TaqMan [38] real-time PCR assays, T2*Candida auris*™ Panel RUO assay [47], and LAMP assay [48] performance have already been validated on panels of clinical swabs. Even though the swabs harbored other microorganisms present in the skin flora, no cross-reactivity was observed, further confirming the high specificity of the assays and their readiness to be applied in real-life surveillance for *C. auris*. 

DNA from both live and dead *C. auris* cells can be detected in molecular-based assays, meaning that swabs stored for a long time can be retrospectively evaluated for the presence of *C. auris*. Several month-long storage of swabs dramatically reduces the viability of *C. auris* cells and chances for their recovery in the enrichment broth. On the other hand, at such a point, *C. auris* DNA is often still present and can be detected by real-time PCR assay [31].

Application of molecular methods for swabs screening can provide a sample flow triage to help offload clinical laboratories from the burden of swab processing. Since only an aliquot (100–200 µL) of the total 1 ml of swab medium is used for DNA isolation, the remaining volume can be stored until the diagnostic result is obtained. PCR/real-time PCR assays indicate *C. auris*-negative samples, so in situation when *C. auris* colonies are still required for further studies, these swabs can be automatically excluded from further processing in the enrichment broth.

We acknowledge that some laboratories still find technical and financial difficulties in introducing molecular methods to their laboratories. However, they can be easily overcome through training and collaboration, and the ever-increasing availability of cost-effective solutions (equipment, reagents) for molecular diagnostics.

## 3. Assessment of *Candida auris* Antifungal Drug Resistance

### 3.1. Antifungal Susceptibility Testing

Antifungal susceptibility testing (AFST) for *C. auris* has mostly been performed using Clinical and Laboratory Standards Institute (CLSI) and European Committee on Antimicrobial Susceptibility Testing (EUCAST) broth microdilution methods [8,9,17,50,51,52]. However, despite standardized methodologies, in vitro susceptibility testing has several limitations that cannot be ignored:

- The assay requires a colony of *C. auris* isolate (widely discussed in 2.1).
- The assay has a time-consuming setup consisting of the following steps: (1) reagents (RPMI, drug stocks) preparation; (2) drug dilutions preparation and their transfer to 96-well plates; (3) standardized number of cells suspension preparation and its inoculation into 96-well plates.
- The assay has intrinsically slow turnaround time, requiring at least 24 h after isolate recovery to allow for the results readout.
- Reading of the AFST results requires some level of expertise, since echinocandin Eagle effect (also known as paradoxical growth effect) and azole trailing growth effect complicate *C. auris* MIC reading. Eagle effect, observed in vitro as reduced antifungal activity at high doses of the drug, significantly complicates accurate minimal inhibitory concentration (MIC) readout for caspofungin. However, this effect has no translation into in vivo response of *C. auris* isolates to the echinocandin treatment as was shown in an invasive murine candidiasis model [17]. We speculate that in some studies the presence of an Eagle effect of high intensity, led to a miscategorization of *C. auris* isolates as caspofungin-resistant resulting in a dangerous overestimation of echinocandin resistance (up to 37% [14]) in *C. auris*.
- No antifungal breakpoints have been defined for *C. auris* so far. Provisionally, CDC provides guidance for *C. auris* MIC interpretation, that is based on information gathered for related *Candida* species and expert opinion (www.cdc.gov/fungal/candida-auris/c-auris-antifungal.html). Moreover, tentative epidemiological cut off values (ECVs) for Indian isolates were determined by Arendrup et al. [8]. The ECVs separate a population of isolates into those with or without mutational resistance based on their phenotypic MIC value. However, since MIC distributions can be quite different among *C. auris* isolates representing different geographical clades [50,53], application of South Asian *C. auris* ECVs to isolates from other clades should be viewed cautiously, as this may lead to incorrect estimation of potential drug resistance.

While standardized susceptibility testing remains the current “gold-standard,” it is important to recognize that analysis of susceptibility results obtained by phenotypic tests is time-consuming and not always straightforward, which (in some hands) leads to difficulties in defining susceptible or resistant isolates.

### 3.2. Molecular Methods

To overcome the slow detection of resistance, it is possible to apply molecular tools since sequence data are highly informative and accurate. DNA sequencing has been the predominant way to identify mutations conferring resistance. However, targeted gene sequencing is impractical for the regular workflow of clinical microbiology laboratories due to its cost, technical demands, and lengthy turnaround times for those without in-house sequencing capacity [22]. Molecular-based rapid tests have the potential to become part of the clinical laboratory routine for resistance detection to help direct therapy and enhance epidemiological surveillance since they allow for analysis of the DNA recovered directly form swabs.

Molecular determinants of azole and echinocandin resistance in *C. auris* have been investigated by several groups. *FKS1* S639P [54] or S639F [9,17] and *ERG11* F126L, Y132F, or K143R [16,55,56] are the only mutations clearly linked to the clinical failure due to azole and echinocandin resistance, respectively. To date, the molecular mechanism(s) of *C. auris* amphotericin B resistance have not been elucidated.

The application of molecular-based allele-specific probe technology is widely used for pathogen detection and assessment of drug resistance (e.g. Xpert MTB/RIF.) Molecular beacons, small stem-loop-structured DNA oligonucleotides, in conjunction with real-time PCR enable easy differentiation of wild-type (WT) and mutant amplicons based on their melting temperature (T_m_). This approach was initially developed to help assess Cyp51A-related azole resistance in *Aspergillus fumigatus* [57]. It was also successfully applied for typing of *C. glabrata FKS1* and *FKS2* echinocandin resistance-conferring mutations [58] in high-throughput studies [59]. To facilitate *C. auris* drug resistance screening, a duplex *ERG11* assay enabling detection of mutations at positions Y132 and K143, and a simplex *FKS1* HS1 assay enabling detection of mutations at position S639 were developed. Results from the assays were obtained in 2 hours and were 100% concordant with DNA sequencing results [60].

The assay setup and readout do not require special expertise since the thermocycling parameters can be saved and readily available anytime assay needs to be performed, and the interpretation of the results is straightforward since the testing strain can be easily categorized by comparing its T_m_ value with those generated by WT and mutant reference strains. The assays have a multiplex potential by combining several probes and expandable readiness for incorporating novel mutation detection, by simply adding the corresponding genotype in the reference library without the need for assay redesign [60].

Molecular resistance determinants detection assays can be applied for high-throughput surveillance screening and provide information on *C. auris* isolates susceptibility much quicker than standard methods (especially when performed with DNA isolated directly from clinical swabs). Thus, they have the potential to overcome the deficiencies of existing culture-based assays and improve the availability and accuracy of information on *C. auris* antifungal drug resistance to guide infection prevention and control actions and selection of treatment regimens.

## 4. Conclusions

The future of *C. auris* diagnostics is bright with new molecular assays and technologies being developed each year. We have remarkable diagnostic advancements at our fingertips, which can substantially enhance detection capacity and facilitate outbreak response. With quicker and more efficient diagnostics, clinicians can make more informed treatment decisions (e.g., limit the use of broad-spectrum antimicrobials, minimize chances for the development of resistance), and infection control specialists are able to timely introduce infection prevention and control actions.
